# Liquid-assisted grinding and ion pairing regulates percentage conversion and diastereoselectivity of the Wittig reaction under mechanochemical conditions

**DOI:** 10.3762/bjoc.14.57

**Published:** 2018-03-23

**Authors:** Kendra Leahy Denlinger, Lianna Ortiz-Trankina, Preston Carr, Kingsley Benson, Daniel C Waddell, James Mack

**Affiliations:** 1 Department of Chemistry, University of Cincinnati, PO Box 210172, Cincinnati, OH 45221-0172, USA

**Keywords:** green chemistry, high-speed ball milling, HSBM, LAG, liquid-assisted grinding, Wittig

## Abstract

Mechanochemistry is maturing as a discipline and continuing to grow, so it is important to continue understanding the rules governing the system. In a mechanochemical reaction, the reactants are added into a vessel along with one or more grinding balls and the vessel is shaken at high speeds to facilitate a chemical reaction. The dielectric constant of the solvent used in liquid-assisted grinding (LAG) and properly chosen counter-ion pairing increases the percentage conversion of stilbenes in a mechanochemical Wittig reaction. Utilizing stepwise addition/evaporation of ethanol in liquid-assisted grinding also allows for the tuning of the diastereoselectivity in the Wittig reaction.

## Introduction

**Figure 1:**
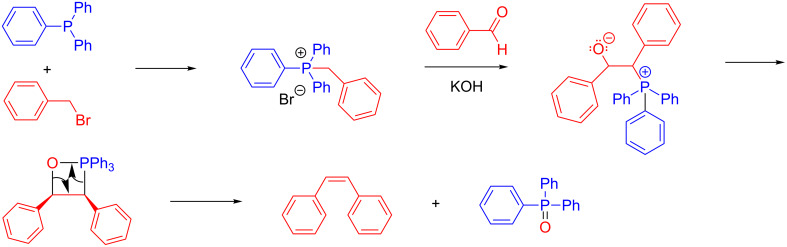
Solution-based Wittig reaction mechanism.

Mechanochemistry is maturing as a discipline and continuing to develop and grow [1–16]. Thus it is important to continue studying and understanding the rules governing the system. Under mechanochemical conditions, the reactants are added into a vessel along with one or more grinding balls, and the vessel is shaken at high speeds to generate the product. Several years ago, Balema and Percharsky first demonstrated the success of the Wittig reaction under mechanochemical conditions [17–18]. The Wittig reaction is one of the most useful reactions for the synthesis of olefins [19–23]. Aside from its synthetic utility, its unique reaction mechanism (shown in Figure 1) and inherent diastereoselectivity has led to a vast amount of intrigue by the chemical community [24–26].

Our research group has continued the study of the Wittig reaction under mechanochemical conditions with the use of a functionalized polymer resin. During these studies, we discovered a few exciting differences between our results and the ones obtained under traditional solution-based conditions. First, we observed that using functionalized resins allowed us to isolate the desired product in an easy and environmentally benign manner. Second, we observed that the incorporation of liquid-assisted grinding (LAG) increased the rate of the reaction in comparison to completely solvent-free conditions. Finally, we observed that there was an effect of the dielectric constant of the solvent used in LAG on the stereochemistry of the product [27]. Although previously we were able to generate high yields of Wittig products under liquid-assisted grinding conditions, we did not truly understand the influence of the reaction medium on the reaction. Therefore, we were interested in better understanding these observations, with the goal of increasing the overall conversion and having better control over the stereoselectivity of the product.

## Results and Discussion

### Liquid-assisted grinding

To focus the study, benzaldehyde, benzyl bromide, polymer-supported triphenylphosphine (PS-C_6_H_4_-PPh_2_) and potassium carbonate were ball-milled in a stainless steel vial with two LAG solvents at opposite ends of the dielectric spectrum, as well as a control without any solvent (Table 1).

**Table 1:** LAG solvent effect on the mechanochemical Wittig reaction.

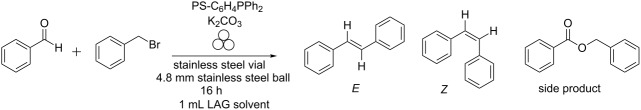				

LAG solvent	dielectric constant [17]	% conversion to stilbene	*E*:*Z* ratio	stilbene/side product ratio

none	–	30	67:33	1/0.83
toluene	2.38	25	61:39	1/0.44
ethanol	24.5	95	40:60	1/0.03

In general, we noticed that more polar solvents (high dielectric constants) favour *Z* selectivity and a higher overall conversion, whereas the use of less polar or no solvent (lower dielectric constants) favour *E* selectivity and a lower overall conversion.

**Figure 2:**
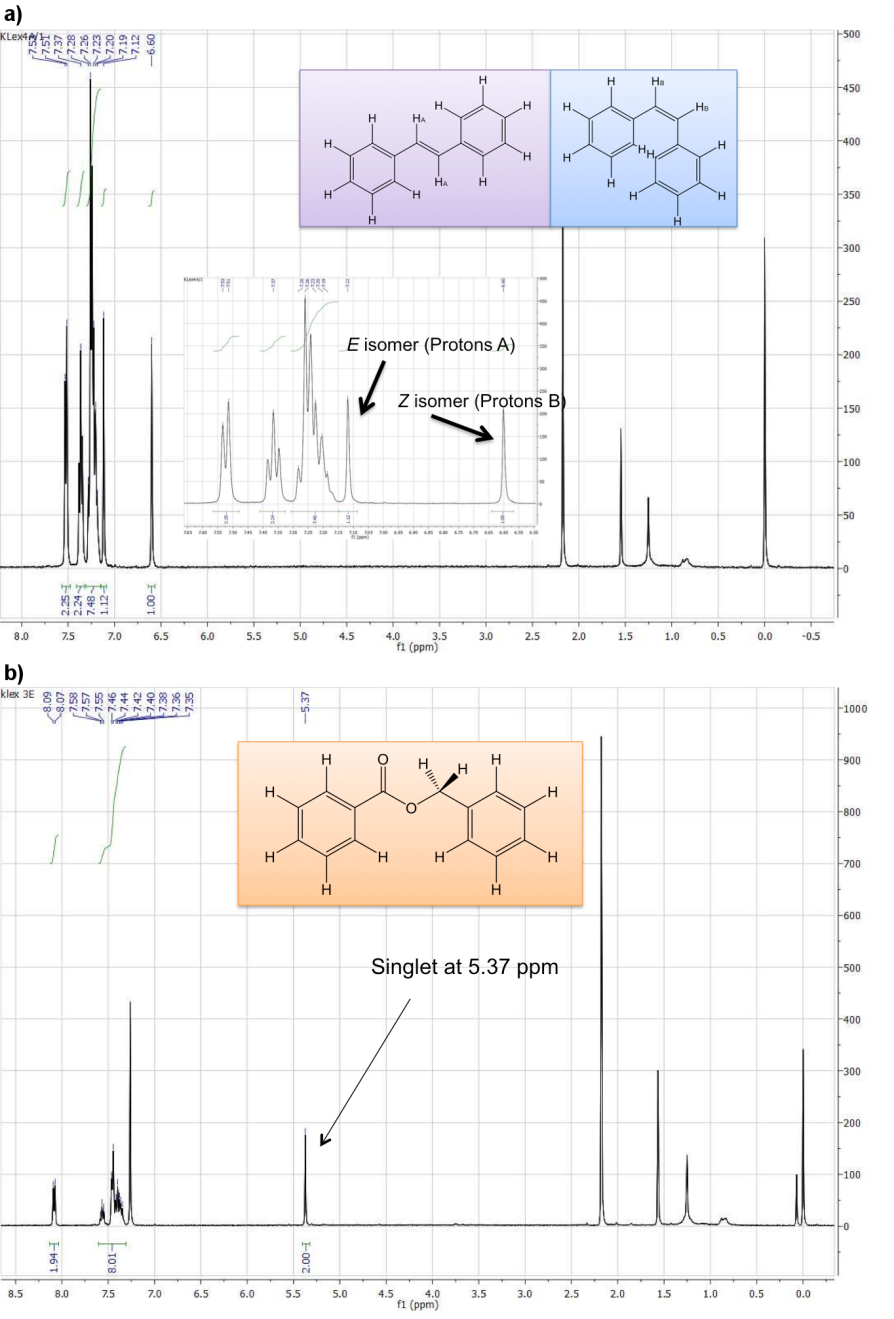
^1^H NMR spectra of stilbene mixture (a) and benzyl benzoate (b).

As shown in Table 1, a side product (benzyl benzoate) is also generated during the reaction. ^1^H NMR spectroscopy was used to determine the *E*:*Z* ratio of the product as well as the percentage of the side product formation. Figure 2 shows the particular peaks for each compound integrated to determine product ratios.

It is important to note that under traditional solution-based conditions, benzyl benzoate has never been reported as a product in the Wittig reaction. Under mechanochemical conditions, the side-product formation appears to be hindered when utilizing a LAG solvent with a high dielectric constant. These results were used to probe the ability to tune the Wittig reaction under mechanochemical conditions.

To determine the origin of benzyl benzoate, we performed a number of control reactions to determine if all reactants are necessary to form the side product (Table 2). Benzyl bromide was absent in each control reaction, and a “√” in the table indicates reactants that were present in each control trial.

**Table 2:** Control reactions to determine the origin of the side product benzyl benzoate.

control trial	benzaldehyde	K_2_CO_3_	PS-C_6_H_4_-PPh_2_	result

1	√			No reaction
2	√	√		No reaction
3	√		√	No reaction
4	√	√	√	No reaction

**Scheme 1:**
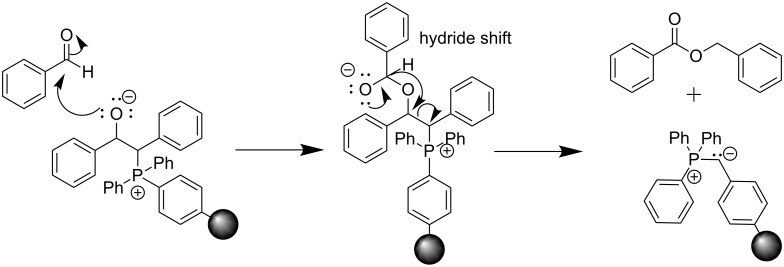
Possible mechanism of benzyl benzoate formation.

Interestingly, it was found that all four components of the reaction (benzyl bromide, benzaldehyde, base, and PS-C_6_H_4_-PPh_2_) are necessary for the production of benzyl benzoate. Based on this evidence, we propose a mechanism for the formation of this side product, which involves the addition of benzaldehyde to the traditional betaine intermediate of the Wittig reaction. This addition step occurs before the rotation and formation of the oxaphosphetane (Scheme 1). In the presence of very small amounts of solvent (LAG) or no solvent at all, the concentration of reactants is very high. This could cause the additional benzaldehyde to be close enough to the intermediate to react before the rotation occurs. To bolster further this argument, the highest amount of side product is observed in the absence of solvent, i.e., at highest reactant concentration (Table 1).

**Scheme 2:**
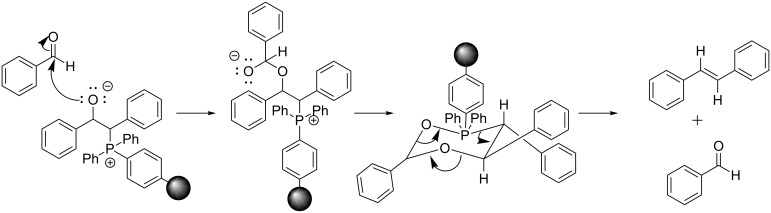
A possible mechanistic explanation for the *E* selectivity.

Further, our results show that the *E* selectivity and benzyl benzoate formation are observed together (when the dielectric constant of the LAG solvent is low). Therefore, if the benzyl benzoate formation follows the path presented in Scheme 1, perhaps the mechanism of the *E* selectivity is similar. The intermediate (Scheme 1) allows for a reaction pathway involving the formation of a six-membered ring instead of the traditional four-membered oxaphosphetane ring of the Wittig reaction. This six-membered ring may account for the higher *E* selectivity due to the preference for larger groups to be in equatorial positions in cyclohexane rings (Scheme 2).

**Scheme 3:**
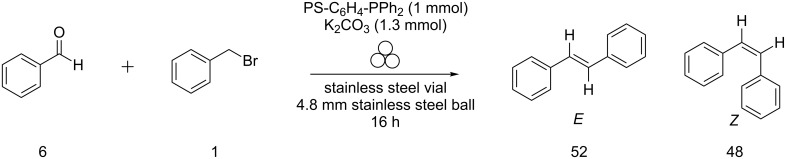
Ball-milled Wittig reaction using excess benzaldehyde.

The *E* selectivity is driven by both the high concentrations of reactants and a low dielectric constant of the LAG solvent (or no solvent). To evaluate which might be playing a more critical role in the selectivity, the reaction was run with an excess of benzaldehyde (Scheme 3) to increase the concentration of reactants. At the same time, benzaldehyde can be considered a LAG solvent with a high dielectric constant (benzaldehyde has a dielectric constant of 17.8 [28]). The increased concentration should favour *E* selectivity, but the high dielectric constant should favour Z selectivity.

Compared to the original reaction (*E*:*Z* ratio 67:33, Table 1, entry 1), the reaction with excess benzaldehyde resulted in an increase in *Z* selectivity with an *E*:*Z* ratio of 52:48. Therefore, it can be concluded that, if a LAG solvent is present, its dielectric constant will be the determining factor in diastereoselectivity, consistent with our previous observations.

### Counter-ion pairing

**Figure 3:**
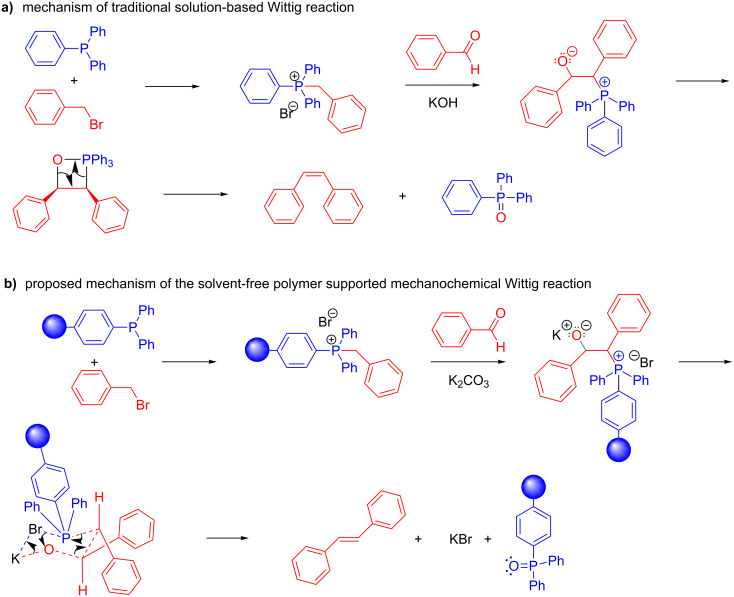
Comparison of solution based Wittig reaction (a) with polymer-supported mechanochemical Wittig reaction (b).

We further hypothesized that, if the benzyl benzoate is formed through a six-membered ring intermediate to give (*E*)-stilbene as the major product, then the same rationale could be used in the case of our solvent-free conditions. In solution, ions are separated and stabilized by solvent molecules. Mechanistically we envision ions to start out as contact ion pairs, then solvent separated ion pairs (i.e., loose ion pairs) followed by free ion pairs. However, this pathway is shut down under solvent-free conditions, making everything in the system a contact ion pair. The traditional solution-based mechanism of the Wittig reaction proceeds via a four-membered oxaphosphetane intermediate. However, by incorporating the halide anion and the alkali metal cation into the mechanism, a six-membered ring, similar to that proposed in Scheme 2, would result (Figure 3).

Based on this hypothesis, in addition to the oxygen and phosphorous forming a stable bond, the alkali metal and halide must form a stable contact ion pair as well. These interactions and the formation of this bond may have a large influence under mechanochemical conditions because there is not a solvent reservoir to accept the dispersion of these ions. Using Pearson’s hard and soft acid and base (HSAB) theory [29] and the Jones–Dole viscosity B coefficient [30] (Table 3), we can predict which alkali metal and halide pairs would be most favourable. For example, bromide is a borderline soft anion, so based on the proposed mechanism we would expect more product to form if the counter ion is Cs^+^(soft) than if it was Li^+^ (hard). Using the Jones–Dole viscosity B coefficient, we could also predict that Cs^+^ and Br^−^ would be a good pair, since their values are similar. We would expect Rb^+^ to pair well with Br^−^ for the same reason. To test this idea and to understand the effect of the interaction, we conducted the solvent-free polymer-supported Wittig reaction with various carbonate salts and alkyl halides (Table 4).

**Table 3:** Counter-ion partnerships.

Ion	Pearson HSAB concept	Jones–Dole viscosity B coefficient [31]	Ionic radius (pm)

Li^+^	hard	0.150	76
Na^+^	hard	0.086	102
K^+^	hard	−0.007	138
Rb^+^	borderline	−0.030	152
Cs^+^	soft	−0.045	167
Cl^-^	hard	−0.007	181
Br^-^	borderline	−0.032	196

**Table 4:** Counter-ion partnerships in the solvent-free mechanochemical Wittig reaction.

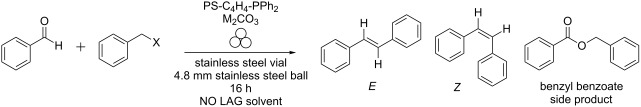					

trial	cation (M^+^)	anion (X^−^)	*E*:*Z* ratio	conversion to stilbene	conversion to benzyl benzoate

1	Li	Br	–	0%	6%
2	Na	Br	–	0%	29%
3	K	Br	67:33	30%	45%
4	Cs	Br	78:22	72%	9%
5	Li	Cl	–	0%	10%
6	Na	Cl	72:28	11%	13%
7	K	Cl	69:31	37%	24%
8	Cs	Cl	74:26	36%	28%

Pairing of a hard acid (Li^+^ or Na^+^) with a moderately soft base (Br^−^) leads to no or poor conversion to stilbene products. Conversely, the best conversion resulted when combining Cs^+^ (soft acid) with Br^−^ (borderline soft base). The benzyl benzoate side-product observed in trials 1, 2, and 5 indicates that the carbonate bases deprotonated the phosphonium salt to form the ylide which then subsequently added to the benzaldehyde. However, the oxygen anion could not bind to the phosphorus cation to produce the stilbene product, presumably due to the mismatched counter ion pair. After identifying the caesium–bromine pair was optimal for the conversion, caesium carbonate was the base of choice for the described stepwise study with ethanol.

### Tuning liquid-assisted grinding with ethanol

Because using ethanol (high dielectric constant) as the LAG solvent afforded the highest conversion to stilbene and the least amount of benzyl benzoate, we began our study on the yield and diastereoselectivity using this solvent. First, we were interested in the influence of ethanol on the mechanochemical reaction of the alkyl halide and the polymer-supported triphenylphosphine. For this purpose, PS-C_6_H_4_-PPh_2_ and 4-nitrobenzyl bromide were ball-milled for two hours with and without ethanol as the LAG solvent. Afterwards, the reaction mixture was filtered with ethanol to determine the amount of 4-nitrobenzyl bromide in solution: the higher the amount recovered means that less 4-nitrobenzyl bromide reacted and is bound to the polymer and thus less production of the desired phosphonium salt. As expected, only 8.5% unreacted 4-nitrobenzyl bromide was recovered when ethanol was used as a LAG solvent, demonstrating that ethanol is an effective LAG solvent for the production of the phosphonium salt (Table 5).

**Table 5:** How much 4-nitrobenzyl bromide adds to the polymer-supported triphenylphosphine, PS-C_6_H_4_-PPh_2_?

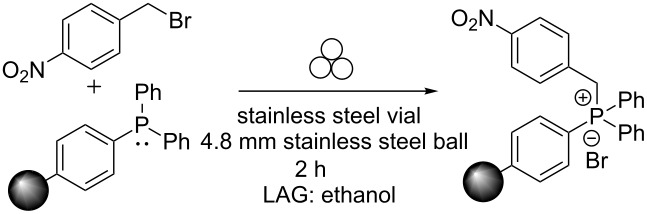	

ethanol as LAG solvent?	percent mass recovery^a^

no	79.5 %
yes (1 mL)	8.5%

^a^Average of two trials.

**Scheme 4:**
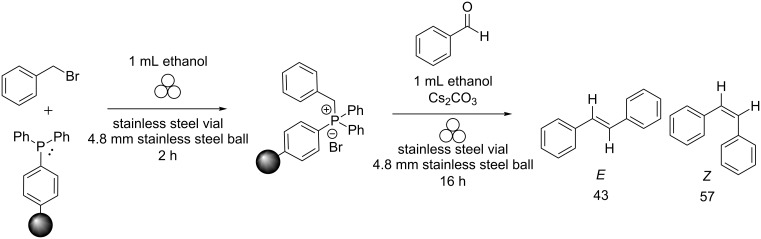
Stepwise ball-milled Wittig reaction with ethanol as the LAG solvent.

Because the formation of the phosphonium salt is the first step of the Wittig reaction, the question arose whether performing the reaction stepwise could influence our ability to select for both percent conversion and diastereoselectivity. Using a stepwise reaction approach with ethanol as the LAG solvent (no work-up performed between the steps), a 98% conversion to stilbene was observed with an *E*:*Z* ratio of 43:57 (Scheme 4).

**Scheme 5:**
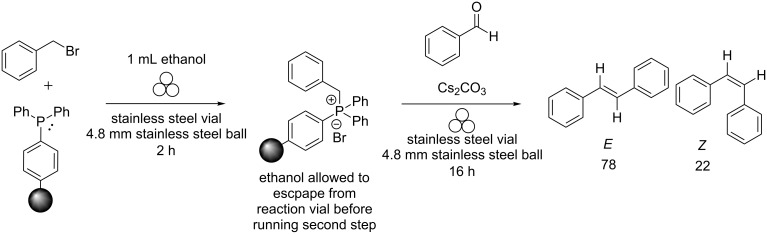
Stepwise ball-milled Wittig reaction with ethanol evaporation between the steps.

As can be seen from the scheme, the reaction proceeded with high conversion and *Z* selectivity, which was ascribed to the high dielectric constant of ethanol. However, if ethanol was allowed to evaporate from the vial before the addition of benzaldehyde, thus creating a non-LAG condition, then *E* selectivity should be favoured for the Wittig reaction. Indeed, it turned out that a 98% conversion to the product along with an *E*:*Z* ratio of 78:22 (Scheme 5) occurred.

As shown, the dielectric constant of the solvent used in LAG can affect both the percent conversion of the reaction as well as the diastereoselectivity. By running the reaction stepwise, we can tune the reaction to proceed with high percent conversion while changing the diastereoselectivity of the product.

## Conclusion

Both a high dielectric constant of the solvent used in liquid-assisted grinding (LAG) and proper ion pairing were found to increase the percent conversion to stilbenes under mechanochemical conditions. Choosing appropriate ion pairs when LAG is utilized in the system also allowed tuning the diastereoselectivity. Specifically, this selectivity could be achieved by combining the Cs^+^/Br^−^ pair with the LAG solvent as follows: if one millilitre of ethanol was present in both reaction steps a higher *Z* selectivity was obtained. If one millilitre of ethanol was present only in the first step of the experiment, a higher *E* selectivity was obtained. The high concentration of reactants under mechanochemical conditions allows for unique and potentially selective reactions that may not be achievable by traditional synthetic means. Further studies on the influence of HSAB theory and the Jones–Dole viscosity B coefficient under mechanochemical conditions are ongoing.

## Experimental

NMR spectra were obtained using a Bruker Avance 400 MHz spectrometer. Deuterated chloroform was obtained from Cambridge Isotope Laboratories Inc., Addover, MA, and used without further purification. Triphenylphosphine-functionalized polystyrene, 2% cross-linked with divinylbenzene (PS-C_6_H_4_-PPh_2_) was obtained from Biotage® and used without further purification. Benzaldehyde was obtained from Sigma Aldrich and used without further purification. Alkyl halides and carbonate bases were obtained from Fisher Scientific and used without further purification.

### Mechanochemical Wittig reaction

To a customized stainless steel vial (3.0 mL volume) was added 1 mmol (500 mg) of PS-C_6_H_4_-PPh_2_, 0.998 mmol alkyl halide, 0.58 mmol aldehyde, and 1.3 mmol carbonate base. This mixture was ball-milled for 16 h. For liquid-assisted grinding experiments, also 1 mL solvent was added. For stepwise reactions, PS-C_6_H_4_-PPh_2_, the alkyl halide, and the LAG solvent were ball-milled for 2 h. Afterwards, the aldehyde and carbonate bases were added, and the reaction mixture was ball-milled for further 16 h. A 4.8 mm (3/16”) stainless steel ball was added to the vial for all steps. The vial was shaken at 18 Hz in a Spex8000M Mixer/Mill. To work up the reaction after the reaction was complete, 2 mL of ethyl acetate were added to the vial, and the vial was returned to the mill for 5 min. The resulting mixture was gravity filtered with ethyl acetate. The solvent was removed under reduced pressure. ^1^H NMR spectroscopy was performed to assess the composition of the filtrate.
